# Mall Walking Program Environments, Features, and Participants: A Scoping Review

**DOI:** 10.5888/pcd12.150027

**Published:** 2015-08-13

**Authors:** Laura Farren, Basia Belza, Peg Allen, Sarah Brolliar, David R. Brown, Marc L. Cormier, Sarah Janicek, Dina L. Jones, Diane K. King, David X. Marquez, Dori E. Rosenberg

**Affiliations:** Author Affiliations: Basia Belza, Sarah Brolliar, Dori E. Rosenberg, Health Promotion Research Center, University of Washington, Seattle, Washington; Peg Allen, Brown School, Washington University in St. Louis, St. Louis, Missouri; David R. Brown, Centers for Disease Control and Prevention; Atlanta, Georgia; Sarah Janicek, David X. Marquez, University of Illinois at Chicago, Chicago, Illinois; Marc L. Cormier, Dina L. Jones, Department of Orthopaedics, Division of Physical Therapy, and Injury Control Research Center, West Virginia University; Diane K. King, Center for Behavioral Health Research and Services, University of Alaska Anchorage. Ms Farren is now affiliated with the Mailman School of Public Health, Columbia University, New York, New York, and Dr Cormier is now affiliated with the Department of Kinesiology and Health Promotion, University of Kentucky, Lexington, Kentucky.

## Abstract

**Introduction:**

Walking is a preferred and recommended physical activity for middle-aged and older adults, but many barriers exist, including concerns about safety (ie, personal security), falling, and inclement weather. Mall walking programs may overcome these barriers. The purpose of this study was to summarize the evidence on the health-related value of mall walking and mall walking programs.

**Methods:**

We conducted a scoping review of the literature to determine the features, environments, and benefits of mall walking programs using the RE-AIM framework (reach, effectiveness, adoption, implementation, and maintenance). The inclusion criteria were articles that involved adults aged 45 years or older who walked in indoor or outdoor shopping malls. Exclusion criteria were articles that used malls as laboratory settings or focused on the mechanics of walking. We included published research studies, dissertations, theses, conference abstracts, syntheses, nonresearch articles, theoretical papers, editorials, reports, policy briefs, standards and guidelines, and nonresearch conference abstracts and proposals. Websites and articles written in a language other than English were excluded.

**Results:**

We located 254 articles on mall walking; 32 articles met our inclusion criteria. We found that malls provided safe, accessible, and affordable exercise environments for middle-aged and older adults. Programmatic features such as program leaders, blood pressure checks, and warm-up exercises facilitated participation. Individual benefits of mall walking programs included improvements in physical, social, and emotional well-being. Limited transportation to the mall was a barrier to participation.

**Conclusion:**

We found the potential for mall walking programs to be implemented in various communities as a health promotion measure. However, the research on mall walking programs is limited and has weak study designs. More rigorous research is needed to define best practices for mall walking programs’ reach, effectiveness, adoption, implementation, and maintenance.

## Introduction

Middle-aged and older adults are the least physically active age groups (1). Routine physical activity is associated with lower risk for several chronic diseases and improves health outcomes (2). The rapidly aging population and growing proportion of adults with chronic diseases heightens the need for more universally accessible walking environments to promote healthy aging.

The built environment influences physical activity (3,4). Features such as traffic and crime, limited number of resting places, and poor lighting affect people’s willingness to walk (5,6). Inclement weather limits walking outdoors (6,7). Neighborhood walking can be challenging for walkers with physical or cognitive impairments (5,8–12). Many people seek well-lit, safe, accessible, indoor venues for walking, such as shopping malls (13).

Malls are indoor or outdoor structures that host retail establishments. In 2003, malls were the second most preferred walking site for physical activity in the United States (after neighborhood streets) (14). Mall walking is a no-cost or low-cost physical activity of choice for many adults (13). There are approximately 109,500 shopping centers in the United States that range in size from small convenience centers to large super-regional malls (15). Despite a decline in the number of malls, effective mall walking programs have potential for widespread public health impact, because many of these spaces are being repurposed for other uses and can still host walking programs (16). However, limited research is available on the reach and effectiveness of mall walking programs, and few studies inform best practices for mall policies, environments, or walking programs (17,18). The purpose of this study was to summarize the evidence on mall walking programs’ reach, effectiveness, adoption, implementation, and maintenance to inform practice and future study.

## Methods

### Data sources

We conducted a scoping review of the literature using the RE-AIM (reach, effectiveness, adoption, implementation, and maintenance) framework (17,18) to determine the evidence on the benefits of mall walking programs. A scoping review differs from other reviews in that it addresses broader topics, may include a larger variety of study designs, and may not assess study quality (19–21). Scoping reviews are useful in areas in which researchers are unable to perform systematic reviews because of a lack of studies that use rigorous designs, such as randomized controlled trials. Arksey (19) developed a 6-stage methodological framework for conducting scoping reviews. Recommendations have been made to clarify the stages; one recommendation is to use an iterative process for selecting studies and extracting data (21). To our knowledge, no scoping reviews have been conducted on the evidence on mall walking programs for middle-aged and older adults. We used an iterative process of Web-based searches and bibliography reviews to identify articles (22) (Figure).

**Figure:**
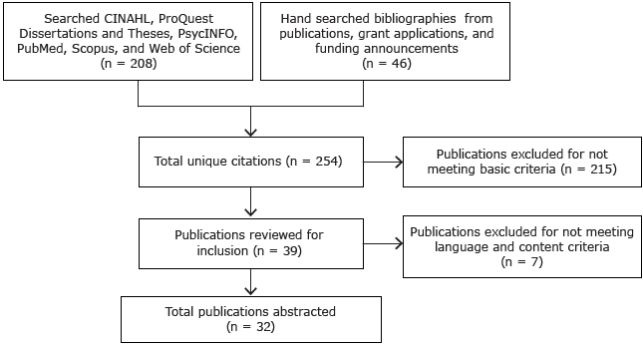
Article search and selection for a scoping review of evidence on mall walking programs for middle-aged and older adults.

We conducted several searches to look for and identify key words, subject headings, and limiters. The most relevant key words were “walk” or “walking” and “mall” or “shopping center,” refined with “middle-aged adults” and “older adults” (≥45 y), “health promotion,” “health behavior,” and “program evaluation.” Using these key words, we searched the following databases: CINAHL, ProQuest Dissertations and Theses, PsycINFO, PubMed, Scopus, and Web of Science. All articles published through December 2013 were searched; we conducted an updated electronic search in April 2015.

A hand search is necessary to comprehensively identify studies in a scoping review, and it involves reviewing key journals for relevant articles (21). Bibliographies from publications, grant applications, and funding opportunity announcements in the field of mall walking for health promotion among middle-aged and older adults were located. These bibliographies were reviewed according to the inclusion criteria. Reference lists of the selected articles from the databases and hand search were reviewed for additional relevant citations.

### Study selection

The inclusion criteria were articles that involved adults aged 45 years or older who walked in indoor or outdoor shopping malls. Originally, the review focused only on adults aged 65 or older, but we expanded the inclusion criteria because many articles focused on both middle-aged and older adults. We did not include other age groups, because few articles mentioned younger walkers. We defined a mall as “a large building, or group of buildings, containing stores of many different kinds and sizes” and is adjacent to one or more parking areas. The stores are connected by indoor hallways or outdoor paths, and are managed together, as one cohesive unit (23). Exclusion criteria were articles that used malls as laboratory settings or focused on the mechanics of walking. We included published research studies, dissertations, theses, conference abstracts, syntheses, nonresearch articles, theoretical papers, editorials, reports, policy briefs, guidelines, conference abstracts, and proposals. Websites and articles written in a language other than English were excluded. Two team members screened the articles. They sent the included articles to a third team member to review and make a final decision on which articles to include.

### Data extraction

The articles were distributed to 7 dyads of senior investigators and graduate students. Dyads read their assigned articles and assumed a primary or secondary role in the abstraction process. The primary reviewer abstracted information from the article. The secondary reviewer evaluated the completed abstraction and noted areas of disagreement. Teams reconciled the abstraction information and entered a final version into an online abstraction tool, which was created by the authors using an online survey generator through the University of Washington. The articles were abstracted for the following: type of article; purpose; name of walking program; setting; population; walking program features; feasibility, acceptability, efficacy, and effectiveness; barriers; cost; information for underrepresented groups; adaptations; cultural relevance; shifting trends; study limitations; and gaps in research. Results were compiled in both Excel and PDF data outputs from the online abstraction tool. A subset of team members separated the data into 7 broad categories: benefits, components of effective mall walking programs, cost, culture, environment, facilitators and barriers, and features. The team reviewed the data in those categories and agreed on the conclusions.

The team reviewed the results, based on the RE-AIM framework, which is a model used to evaluate the reach, effectiveness, adoption, implementation, and maintenance of health promotion programs at the individual and organizational levels (17,18). The *reach*, or program utilization, of an intervention is an individual-level assessment of the number or characteristics of program participants in a given population (17,18); this assessment focuses on the involvement of those in at-risk groups, specifically the sociodemographic characteristics of participants. The *effectiveness* of an intervention is an individual-level assessment of whether the intervention altered the health or behavior of individuals in a given population (17,18); this study qualitatively describes the physical, social, and psychological benefits of mall walking at the individual level. The *adoption* of an intervention refers to the organizational-level readiness and ability to host programs (17,18); this review focuses on the key structural factors that influence program adoption at the organizational level. *Implementation* refers to the many organizational-level facilitators and barriers that are involved in initiating an intervention with fidelity to the original model (17,18); in this review, we focus on the key logistical factors involved in mall walking programs and describe several existing models. The *maintenance* of an intervention includes an individual- and organizational-level assessment of program sustainability (17,18); this assessment focuses on the factors that influence program longevity and walker participation.

## Results

The electronic database search yielded 208 articles; 46 articles were located by hand search. Of the 254 articles selected for an initial screening, 215 were excluded. After a second screening, another 7 articles were excluded. Thirty-two articles were included in the final abstraction process. No additional articles were found in the updated search in April 2015.

Most (n = 27) articles (Table 1) were peer-reviewed, published, and experimental or nonexperimental research studies. All (n = 32) focused on mall walking programs in the United States. The nonexperimental articles (n = 22) described cross-sectional studies (n = 9), case studies (n = 3), descriptive studies (n = 4), qualitative studies (n = 3), comparative effectiveness studies (n = 2), or a qualitative synthesis/theoretical study (n = 1). The experimental articles (n = 5) described quasi-experimental studies (n = 3), a randomized control trial (n = 1), or a single subject (n = 1). Five articles were not research based: 2 reports, 1 editorial, 1 exercise advice column, and 1 opinion piece. Most articles discussed a specific mall walking program (n = 20), while the remaining (n = 12) discussed mall walking in general or as part of a multifaceted exercise approach. Nineteen articles focused on indoor walking, 1 article focused on outdoor walking, 5 articles addressed indoor and outdoor walking, and 7 articles did not specify whether indoor or outdoor.

**Table 1:** Studies on Mall Walking (1983–2012), Scoping Review of the Environments, Features, and Participants of Mall Walking Programs, 2015^a^

Author (Year)	Design, Sample, Methods, Country	Program Features	Facilitator (F) or Barrier (B)
Bird et al (2010) (24)	Descriptive; n = 333, mean age, 72 y; questionnaire; Australia	None noted	**(F) **Socialize with friends
Blair (1988) (25)	Descriptive; n = 15, mean age, 71 y; questionnaire; United States	Check blood pressure and weight, speakers, food, free subscription to *Walking Magazine*	**(F)** Safe, controlled temperature, pleasant environment, indoors, no threat of unattended dogs
Choi et al (2007) (26)	Descriptive, case study; n = 4; pedometer; Canada	None noted	None noted
Colwell (2010) (27)	Descriptive, case study; n = not available; adults on a limited, fixed income; United States	None noted	**(F)** Funding secured from donations, positive health and social outcomes (**B)** cost to participate
Cresswell (2009) (28)	Editorial; Canada, United Kingdom, United States	Check blood pressure, warm-ups, theme days, t-shirts, leaders, routes, mileage markers, awards, coupons, discounts, Mall Walkers Den	**(F)** Security, restroom facilities, well-lit, membership club
Culos-Reed et al (2008) (29)	Experimental, single subject; n = 52, mean age, 66.4 y; self-report, objective measures; Canada	None noted	**(F)** Decrease in body mass index and waist-to-hip ratio; improved flexibility; lower rate of perceived exertion, resting heart rate
Denny (1988) (30)	Cross-sectional; n = 62, mean age, 70 y; questionnaire; United States	None noted	None noted
Duncan et al (1995) (13)	Descriptive; n = 14, age range, 61–81 y; participant observations, interviews; United States	Blood pressure checks, health education programs	**(F)** Expert advice; goals; invitations; substitute for roles and routines lost through retirement; community
Duncan et al (1994) (31)	Theoretical article; United States	Blood pressure checks, health education programs	**(F)** Build self-image and independence, coping skills, roles, community; reduce vulnerability
Eves et al (2008) (32)	Quasiexperimental; n = 18,257; observational; China	None noted	**(B)** Hilly landscapes, traffic, humidity barriers to active transport; signs to take stairs ineffective
Eyler et al (2003) (14)	Cross-sectional; n = 1,816, age range, 18–65 y or older; telephone survey; United States	None noted	**(F)** Mall location in neighborhoods make them easy to access
Fletcher and Macauley (1983) (33)	Report; n = 5, age range, 61–64 y; in-person interview; United States	None noted	**(F)** Distance marked, safe, climate controlled, peer support, heated, air conditioning, seats, restrooms, refreshments, carpeting
Gavin (1997) (34)	Cross-sectional; n = 157; survey; Canada	Log book, mall distance calculated, volunteers, breakfasts, incentives, rewards, beaded “lap counters”	**(F)** social aspects, health and fitness, weight loss **(B)** no ride, injury, illness, too busy
McCormack et al (2008) (35)	Cross-sectional; n = 1,474, age range, 18–59 y; survey; Australia	None noted	**(F)** Proximity of destination, associated with walking for transport
Michael et al (2006) (36)	Cross-sectional; n = 105, age >65 y; audit, questionnaire; United States	None noted	**(F)** Availability of utilitarian destinations (malls) was associated with walking
Moore (1989) (37)	Report; n = 150, age >50 y; telephone survey; United States	Blood pressure, glucose and cholesterol screening, educational programs, weight control class, social events, nurse consultations, student training, logbook, badges, materials	**(F)** Physical and mental health, financial benefit for vendors and mall
Owens (2007) (38)	Cross-sectional; n = 128, age range, 40–87 y; United States	Considered community service programs in partnership with hospitals and senior centers for older adults at risk for disability or death	**(F)** Mall walkers had high self-care agency and health-promoting lifestyle
Prohaska et al (2009) (11)	Cross-sectional; n = 884, age >65 y; interviews, objective measures; United States	None noted	**(F)** People with lower Mental Alternation Test scores more likely to walk in malls and less in parks compared with those with higher scores
Rainey (1992) (39)	Randomized clinical trial; n = 132, mean age, 64 y; questionnaire: United States	None noted	**(F)** Mall walkers compared with non-mall–walkers support more the benefits of exercise
Schacht and Unnithan (1991) (40)	Descriptive; n = 50–150 (range); questionnaire, observational, interviews; United States	Mall walking route, locker, mall-walking button, charts to record distances	**(F)** Couples could walk together, social reasons, helps to manage weight, companionship, enjoyment, safe, weather not an issue
Schlapman (1994) (41)	Cross-sectional; n = 438; questionnaire: United States	None noted	**(F)** Mall walkers influenced by a positive perception of health status and beliefs about walking outcomes
Sommers et al (1995) (42)	Cross-sectional; n = 123, mean age, 66 y; questionnaire: United States	None noted	**(F)** Feeling better, gets a day off to a good start, meet people, better health
Stamford (1994) (43)	Exercise advice column; United States	Start a program: find a sponsor, plan 3 days per week, advertise. Start walking: locate program, check with doctor, locate comfortable shoes, establish routine, social support, keep it fun, go at own pace, walk after meal, avoid long strides, monitor medications.	**(F)** Health benefits such as lowering blood pressure, slowing the aging process, maintaining bone health; health promotion
Standefer (1992) (44)	Cross-sectional; n = 75, aged >50 y; observation, interview: United States	None noted	**(F)** Socialization, support networks (eg, partners, children, doctors) is determinant of mall walking, positive association with exercise and health
Stathi et al (2012) (45)	Cross-sectional; n = 25, aged >70 y; interviews, logs, questionnaires, accelerometers; United Kingdom	Amenities (not specified)	**(F)** Access to amenities, positive physical activity perceptions, existing habit of being active, indoor (**B)** Functional limitations, lack of motivation, not having a companion
Stephenson et al (2007) (46)	Quasiexperimental; n = 24, mean age, 60 y; fitness tests; Canada	None noted	**(F)** Improvements in physical activity, body mass index, waist-hip ratio, walk distance; safe, social support
Travis et al (1996) (47)	Feature article (no data); United States	None noted	**(F)** Routine of mall walking, social relationships
Van Puymbroeck et al (2008) (48)	Comparative effectiveness; n = 5, mean age, 59 y; questionnaires, fitness tests; United States	None noted	**(F)** Improved upper-body strength; low-cost, low-impact, and little-equipment required
Van Puymbroeck and Hsieh (2010) (49)	Comparative effectiveness; n = 18, mean age, 59 y; questionnaires, fitness tests; United States	None noted	**(F)** Increased psychological well-being; inexpensive and low-risk activity
Waggener (1993) (50)	Descriptive; n = 94, age range, 21–79 y; objective measures, questionnaire; United States	None noted	**(F)** Positive effect on cardiovascular health
Warin et al (2008) (51)	Case study; n = 1, aged >50 y; field notes, interviews, observations; Australia	$2 fee per session, name badge, shirt, certificate of achievement, birthday parties, photographs taken by management their newsletter.	**(F)** Safe with security, flat, controlled climate, restrooms, no tripping hazards, companionship, routine (**B)** Cost
Zenk et al (2009) (52)	Quasiexperimental; n = 252, age range, 40–65 y; objective, adherence, analysis of relationship between environment and adherence to walking; United States	The presence of a mall within 5 miles of walker’s neighborhood is associated with 44% better walking adherence (compared with no presence of a mall within 5 miles).	None noted

a Studies are listed alphabetically by first author name.

### Reach: walker participation

Four articles described the average mall walker as white, aged in the mid-sixties, and having a high school degree or equivalent (31,38,41,50). Three articles found the mean age of mall walkers to be 70 or older (24,25,30). Other articles described walkers between the ages of 18 and 87 (13,14,29,33,35–37,45,46,48,49,51,52). One case study included a sample of adults on a limited, fixed income (27).

Three articles found that individual-level facilitators to mall walking initiation were a physician’s recommendation, a social invitation, or interest in reaching health goals (31,38,50). Two articles found that individuals joined a walking program for the no-cost or low-cost exercise option, companionship, and atmosphere (34,40). One descriptive study found that older men were more likely to begin walking as a result of a physician’s recommendation or at the urging of their female partners, while older women reported walking for social reasons, including ensuring their male partners exercised (40). One cross-sectional study found that participants started mall walking in an attempt to delay the aging process (30). A descriptive study explained that many began walking in the mall because of the safe and climate-controlled environments (25).

Several individual-level facilitators to continued mall walking participation were reported. Mall walking programs were described as noncompetitive, sociable, and friendly, and walkers did not feel judged on speed, attire, or physique (25,40). In 3 articles, participants reported feeling less fearful and vulnerable when walking in climate-controlled malls (31,40,44). One study noted that malls lacked the threat of unattended dogs (25). Older adults enjoyed exercising in the mall, rather than in health clubs (40). Walkers chose malls over other walking venues because of the attractive features (14). One randomized clinical trial that used an attribution-retraining instrument (a survey that had exercise-related items, including experience with exercise, feelings about exercise, and perceptions of other people’s exercise, and that mall walkers completed by hand after walking in the mall), which strengthened positive beliefs about exercise and increased adherence to walking by older adults (39).

Studies described lack of transportation (34), injury and illness (34), lack of a walking companion (45), and scheduling conflicts (34) as barriers to mall walking participation. One article addressed dropout rates and found that 69% of walkers adhered to the program and 31% dropped out; the most common reason for dropout was “being too busy” (34). In a case-based approach, individual-level fees were also barriers to participation (47,51). One study (28) found that malls, compared with other settings, provided little variation in surroundings. The repetitive scenery was described as a barrier, as were culinary temptations and lack of appropriate footwear for hard surfaces (28). A study focusing on African American women found that these women believed participation would increase if malls organized transportation and expanded walking hours (52). One case study found that individual-level fees imposed a barrier to participation in some low socioeconomic settings (51).

### Effectiveness: walker benefits

Physical health benefits of mall walking were reported in 2 studies (29,46). After an 8-week walking program, participants showed reduced body mass index (BMI), decreased waist-to-hip ratio, increased flexibility, decreased rate of perceived exertion, and decreased resting heart rate compared with baseline. Baseline quality of life was high for participants and remained high at follow-up (29). A mall walking intervention study, using a quasi-experimental design, showed that BMI, waist-to-hip ratio, rate of perceived exertion, and heart rate improved at 12 weeks compared with baseline (46).

Several articles explored the social benefits of mall walking. In 3 studies, walkers felt a sense of community with other walkers (24,34,42) and often met outside of walking hours to socialize (31,51). One study (44) reported that female walkers tended to wear exercise attire and walking shoes, enabling walkers to identify and acknowledge one another in the mall. Another study (34) explained that “although health and fitness reasons remained a priority, mall walkers reported social reasons and feeling good as contributors in the maintenance of their participation.”

Three articles examined the psychological benefits of mall walking (31,42,47). A descriptive study (31) cited participants who reported “mental stimulation, uplifting moods, stress reduction, relaxation, and mental clarity following the activity.” Another study (47) also described the sense of well-being that older adults experienced. Of the 123 participants in a descriptive study (42), 98% reported feeling better after mall walking.

### Adoption: mall participation

Several articles reported mall walking programs were hosted in single-story (31,51) or multiple-story shopping centers (32) with controlled temperatures and limited exposure to bad weather (25,31,45,47,51). Organizational-level facilitators to hosting mall walking programs were reported, including well-lit walkways (24,28), accessible restrooms (28,51), even walking surfaces, safe environments (25,28,41,51), background music, and coffee shops (31). Other studies reported organizational-level facilitators such accessibility (14,35) and availability (36) of malls as walking venues, availability of seats, and carpeting (33). Walking programs were reported to operate before mall business hours, as early as 7:00 AM, 2 to 5 days per week, in 4 articles (28,31,40,51). Malls were reported to open specifically for walking from 1 hour to 4 hours per day in 6 articles (28,32,42,45,47,50). One editorial described a program that was seasonal, operating September through April to avoid the cold weather (28).

### Implementation: program staff, materials, events, and costs

Many mall walking locations hired volunteers or staff from partnering organizations to manage the programs. Six articles reported that these people included fitness instructors and nurse coordinators (25,28,34,48,49,51). In programs described in 3 articles, volunteers managed programs when walking leaders were absent (28,33,34).

Mall walking programs that allowed sites to accommodate diverse populations were described as adaptable in 4 articles (24,32,51,52). One study (24) reported that mall walking program adaptation is necessary, because people of various cultures view walking differently (ie, some view walking as an activity for health and fitness; some view it as leisure activity; and some view it as a destination-oriented activity). One study (40) found that walkers adapted programs to fit their needs, such as allowing people of varying ability levels to participate. One case study described a program that adapted its walking route by providing participants access through a side door while the mall underwent refurbishments (51). Another article described a mall that postponed mall walking start times to clean the floors (31).

Some programs assisted participants in attaining their fitness goals by enabling them to track their progress and measure health metrics. Five articles described programs that offered services such as blood pressure checks, cholesterol screenings, blood glucose screenings, weight control classes, and nurse consultations (25,28,31,37,47). Two articles described programs that provided health education materials (28,32), and 3 articles described programs that provided measurements of walking route distances (27,28,34). Four articles described walking and supplemental fitness programs that calculated distances and distributed maps that showed the mileages of walking routes (27,28,34,40). Three articles described programs that were led by fitness instructors and included opening stretches, resistance band strength exercises, and balance exercises (48,49,51).

Malls provided facility supports, including meeting areas, coat racks, lockers, and security personnel, which served as facilitators to implementation, in programs described in 3 articles (28,34,47). Many programs incentivized walkers to participate by offering matching t-shirts (28,51), activity logs (37,40), magazine subscriptions (25), or mall retailer discounts (28). Two programs used newsletters to promote mall walking (43,51), one of which included photographs of the walkers (51). Several articles described safety (25,28,33,40,46,51) and accessibility (14,35), controlled temperatures (25,33,40,51), available restrooms (28,33,45,51), well-lit walking routes (28), and available refreshments (33) as facilitators to program implementation.

Ten articles described socialization as a facilitator to implementation of mall walking programs (24,27,31,34,40,42,44,46,47,51). Three articles described programs that marketed the social aspect of mall walking and developed program names to emphasize that element (27,34,51). One program hosted a Mall Walkers Den, or private room, for socializing (28). Two programs offered certificates and awards to walkers (27,51), and 4 programs hosted events to encourage social engagement (25,28,34,51). One article (51) described a site that organized a birthday celebration for each walker.

The organizational-level costs of mall walking programs (Table 2) were described as variable and dependent on the program’s features, including staff, materials, and events in 3 articles (27,34,47). To cover these costs and to lessen the financial burden on participants, programs in 5 articles used financial partnerships between malls and businesses, hospitals, community organizations, and individuals to facilitate implementation (27,34,37,44,47). Mall walking is typically a no-cost or low-cost exercise option. Eight articles described programs with individual-level fees, which ranged from $1 per day to $15 per year and were barriers to participation (13,27,28,33,37,40,48,49).

**Table 2:** Costs of Mall Walking Programs (1983–2012), Scoping Review of the Environments, Features, and Participants of Mall Walking Programs, 2015

Author (Year)	Individual-Level Costs	Organizational-Level Costs
Colwell (2010) (27)	$15 per year; $1 per class	Instructor fees, coordinator stipends, materials, resistance bands
Gavin (1997) (34)	None noted	Materials such as log books, incentives, rewards
Moore (1989) (37)	Nominal fee to join the program. Small fee for blood cholesterol screening. Small fee for weight control class.	Costs associated with conducting screenings, staff time, materials such as logbooks and badges. Mall and hospital shared costs for social events.
Schacht and Unnithan (1991) (40)	None noted	Marking the walking route, lockers, buttons, charts to record distances
Standefer (1992) (44)	None noted	Mall opened 2 hours early every day for walkers; walkers frequent the stores and restaurants after their walk; reduced turnover of tables at mall eateries during times when mall walkers are present; mall management accepts this because walkers leave before 11:00 AM.
Travis et al (1996) (47)	None noted	Examination by a physician for approval to walk
Warin et al (2008) (51)	Walkers purchased coffee	Marketing, including producing and distributing leaflets in the mall, through local newspapers, and word of mouth; certificates of completion. Mall management sees the walkers helping to promote mall, mall management attended mall walker events.

### Maintenance: participant and program sustainability

Literature on the maintenance of mall walking programs was limited. However, participants in 3 articles explained that they continued mall walking because of the established routine, as well as the social and emotional benefits (34,38,43). On the organizational level, several articles explored the partnerships among mall walking programs, malls, mall businesses, and the communities in which they operated and found that these partnerships were imperative to program sustainability (27,34,37,44,47,51). Two studies (44,51) described mall walking as mutually beneficial to walkers and mall businesses. One study found that walkers frequented retail establishments and restaurants in the mall and that merchants offered financial discounts to walkers, increasing their exposure and creating relationships between parties (34). A case study reported that mall walking programs gave malls a reputation of social responsibility (51).

## Discussion

The results from this review show that mall walking programs have the potential to increase physical activity among middle-aged and older adults. However, the impact of these programs is unknown. The literature that is available is of low quality. More rigorous research is needed to understand the factors that affect the reach, effectiveness, adoption, implementation, and maintenance of mall walking programs.

Future research must focus on the reach of mall walking programs in diverse communities, using a health equity lens, as well as the potential use of social media to recruit walkers. There is little information in the literature on mall walking program availability in communities of color, in areas of low socioeconomic status, or for people with disabilities or chronic conditions. Although no-cost or low-cost mall walking programs have the potential to reach at-risk and underserved communities because they are in structures where safety is not an issue, additional research is needed to understand the facilitators and barriers to such programs in diverse communities. Social media is a potential tool for expanding program reach.

Future research, using more rigorous study designs, must also focus on the effectiveness of mall walking in improving overall physical activity levels. Several articles described the general physical, social, and psychological benefits of mall walking among participants. However, no studies assessed the exact amount of walking that participants did by using accelerometers, pedometers, or other tracking devices. Given that pedometers, and now smartphone apps, provide feedback and motivation and track walking behavior over time, their use in mall walking programs may be beneficial. The few pilot studies that evaluated anthropometric measures, such as BMI and waist-to-hip ratio, reported positive effects, which suggests that the amount of mall walking done in these studies was sufficient to result in health benefits. The studies included participants with chronic conditions but did not measure condition-specific outcomes. Future studies should explore the issues that predict adherence and nonadherence, as well as how adaptations in the design of mall walking programs may encourage or discourage retention.

Although we reported several facilitators to organizational-level participation, limited data are available on mall walking environments. To implement and maintain effective programs, it is necessary to understand the elements in the built environment that facilitate or hinder walking. Studies are needed to evaluate the built environments of mall walking programs and to understand stakeholder perspectives of these environments to improve recruitment and participation.

As mall walking programs are disseminated more widely, additional research is needed to understand the facilitators and barriers to program implementation. Most malls are safe, indoor facilities that have drinking fountains, seats for resting, level surfaces, restrooms, and proper lighting. However, these features have not been thoroughly evaluated by studies that use rigorous research designs. Mall businesses could benefit from mall walking programs by attracting additional customers and building loyalty and a sense of community among walkers. Although our findings show promise, additional research is needed to better understand organizational-level facilitators and barriers to program implementation.

More rigorous research is needed to understand mall walking program sustainability. Although organizational-level costs may affect sustainability, data on program costs are limited. An economic analysis is needed to understand organizational-level costs, including mall liability costs. Partnerships between malls, local and regional organizations, and universities must be explored, because they may be used to offset program costs through cost-sharing. Partnerships between malls and health-focused organizations, such as hospitals, physical activity facilities, and parks and recreation departments, may provide the mutual benefit of improved health and increased mall visibility in the community.

This study had several limitations. We conducted a scoping review, so the process did not include a registered protocol or a risk-of-bias assessment. The literature on mall walking programs is limited. We included articles published in English only, and our review was limited to articles that focused on adults 45 years of age or older. Many articles were not scientific (ie, reports, editorials, theoretical papers, exercise advice column, feature article, and case studies) (28,31,33,37,43,47,51). The studies we did include were primarily descriptive, had small sample sizes (29,30,36,44,48,49), and followed mall walkers for a short duration (26,29,31,39,46,48,49). The quality of the research designs was not assessed. Despite these limitations, our comprehensive review captures data on what is known about mall walking programs to date and identifies gaps in the literature.

Future research could use the RE-AIM framework to 1) explore the built environment aspects of mall walking, including the facilitators and barriers to program effectiveness; 2) investigate the effectiveness of mall walking programs in diverse community settings; 3) evaluate the efficacy and effectiveness of mall walking programs in increasing overall physical activity in older adults; 4) explore effective adoption, implementation, and maintenance features of mall walking programs through site observations, and interviews with key informants, including a comprehensive cost-benefit analysis; 5) use social marketing or other communication to raise public awareness of the benefits of mall walking; and 6) assess cultural and demographic needs of target populations and develop adaptable mall walking programs using a health equity lens and inclusion of persons with disabilities.

This scoping review was a formative step in the development of *Mall Walking: A Program Resource Guide*, a management tool, which was designed to encourage shopping malls and other community sites to start or enhance walking programs (53) and is available at www.cdc.gov/physicalactivity/downloads/mallwalking-guide.pdf.

## References

[1] Pleis JR , Ward BW , Lucas JW . Summary health statistics for U.S. adults: National Health Interview Survey, 2009. Washington (DC): National Center for Health Statistics; 2010. 21905346

[2] Vogel T , Brechat PH , Leprêtre PM , Kaltenbach G , Berthel M , Lonsdorfer J . Health benefits of physical activity in older patients: a review. Int J Clin Pract 2009;63(2):303–20. 10.1111/j.1742-1241.2008.01957.x 19196369

[3] Saelens BE , Handy SL . Built environment correlates of walking: a review. Med Sci Sports Exerc 2008;40(7 Suppl):S550–66. 10.1249/MSS.0b013e31817c67a4 18562973 PMC2921187

[4] Saelens BE , Sallis JF , Frank LD . Environmental correlates of walking and cycling: findings from the transportation, urban design, and planning literatures. Ann Behav Med 2003;25(2):80–91. 10.1207/S15324796ABM2502_03 12704009

[5] King D . Neighborhood and individual factors in activity in older adults: results from the neighborhood and senior health study. J Aging Phys Act 2008;16(2):144–70. 18483439 10.1123/japa.16.2.144

[6] Rosenberg DE , Huang DL , Simonovich SD , Belza B . Outdoor built environment barriers and facilitators to activity among midlife and older adults with mobility disabilities. Gerontologist 2013;53(2):268–79. 10.1093/geront/gns119 23010096 PMC3605937

[7] Li W , Keegan TH , Sternfeld B , Sidney S , Quesenberry CP Jr , Kelsey JL . Outdoor falls among middle-aged and older adults: a neglected public health problem. Am J Public Health 2006;96(7):1192–200. 10.2105/AJPH.2005.083055 16735616 PMC1483851

[8] Clarke P , Ailshire JA , Lantz P . Urban built environments and trajectories of mobility disability: findings from a national sample of community-dwelling American adults (1986–2001). Soc Sci Med 2009;69(6):964–70. 10.1016/j.socscimed.2009.06.041 19643522 PMC2759178

[9] Kerr J , Rosenberg DE , Frank L . The role of the built environment in healthy aging: community design, physical activity, and health among older adults. J Plann Lit 2012;27(1):43–60. 10.1177/0885412211415283

[10] Nagel CL , Carlson NE , Bosworth M , Michael YL . The relation between neighborhood built environment and walking activity among older adults. Am J Epidemiol 2008;168(4):461–8. 10.1093/aje/kwn158 18567638 PMC2727277

[11] Prohaska TR , Eisenstein AR , Satariano WA , Hunter R , Bayles CM , Kurtovich E , Walking and the preservation of cognitive function in older populations. Gerontologist 2009;49(Suppl 1):S86–93. 10.1093/geront/gnp079 19525221

[12] Satariano WA , Ivey SL , Kurtovich E , Kealey M , Hubbard AE , Bayles CM , Lower-body function, neighborhoods, and walking in an older population. Am J Prev Med 2010;38(4):419–28. 10.1016/j.amepre.2009.12.031 20307811

[13] Duncan HH , Travis SS , McAuley WJ . An emergent theoretical-model for interventions encouraging physical-activity (mall-walking) among older adults. J Appl Gerontol 1995;14(1):64–77. 10.1177/073346489501400105

[14] Eyler AA , Brownson RC , Bacak SJ , Housemann RA . The epidemiology of walking for physical activity in the United States. Med Sci Sports Exerc 2003;35(9):1529–36. 10.1249/01.MSS.0000084622.39122.0C 12972873

[15] Shopping center facts and stats. New York (NY): The International Council of Shopping Centers; 2015. http://www.icsc.org/research/shopping-center-facts-and-stats. Accessed April 12, 2015.

[16] Here’s what’s becoming of America’s dead shopping malls. Washington (DC): National Public Radio, Morning Edition; 2014. http://www.npr.org/2014/09/10/347132924/heres-whats-becoming-of-americas-dead-shopping-malls. Accessed April 12, 2015.

[17] Glasgow RE , Vogt TM , Boles SM . Evaluating the public health impact of health promotion interventions: the RE-AIM framework. Am J Public Health 1999;89(9):1322–7. 10.2105/AJPH.89.9.1322 10474547 PMC1508772

[18] King DK , Glasgow RE , Leeman-Castillo B . Reaiming RE-AIM: using the model to plan, implement, and evaluate the effects of environmental change approaches to enhancing population health. Am J Public Health 2010;100(11):2076–84. 10.2105/AJPH.2009.190959 20864705 PMC2951937

[19] Arksey H . Scoping the field: services for carers of people with mental health problems. Health Soc Care Community 2003;11(4):335–44. 10.1046/j.1365-2524.2003.00433.x 14629205

[20] Jones CA , Pohar S . Health-related quality of life after total joint arthroplasty: a scoping review. Clin Geriatr Med 2012;28(3):395–429. 10.1016/j.cger.2012.06.001 22840305

[21] Levac D , Colquhoun H , O’Brien KK . Scoping studies: advancing the methodology. Implement Sci 2010;5(1):69. 10.1186/1748-5908-5-69 20854677 PMC2954944

[22] Arksey H , O’Malley L . Scoping studies: toward a methodological framework. Int J Soc Res Methodol 2005;8(1):19–32. 10.1080/1364557032000119616

[23] Shopping malls. Springfield (MA): Merriam-Webster; 2015. http://www.merriam-webster.com/dictionary/shopping%20mall. Accessed January 6, 2015.

[24] Bird SR , Radermacher H , Sims J , Feldman S , Browning C , Thomas S . Factors affecting walking activity of older people from culturally diverse groups: an Australian experience. J Sci Med Sport 2010;13(4):417–23. 10.1016/j.jsams.2009.07.002 19833556

[25] Blair JF . An instrument to ascertain the sociological characteristics of mall walking clubs and social health of their older adult members [doctoral dissertation]. Knoxville (TN): University of Tennessee; 1998.

[26] Choi BC , Pak AW , Choi JC , Choi EC . Achieving the daily step goal of 10,000 steps: the experience of a Canadian family attached to pedometers. Clin Invest Med 2007;30(3):E108–13. 17716548 10.25011/cim.v30i3.1078

[27] Colwell S . Reaching out to the whole community with a comprehensive adults exercise program. ACSM’s Health Fit J 2010;14(2):33–4.

[28] Cresswell J . Retail therapy with mall walking. SportEX Health. 2009;14718154(22):20–1.

[29] Culos-Reed SN , Stephenson L , Doyle-Baker PK , Dickinson JA . Mall walking as a physical activity option: results of a pilot project. Can J Aging 2008;27(1):81–7. 10.3138/cja.27.1.81 18492639

[30] Denny DC . The structured and unstructured activity of older adults as measures of life satisfaction: a comparative study [doctoral dissertation]. Buffalo (NY): D’Youville College; 1988.

[31] Duncan HH , Travis SS , McAuley WJ . The meaning of and motivation for mall walking among older adults. Act Adaptation Aging 1995;19(1):37–52. 10.1300/J016v19n01_03

[32] Eves FF , Masters RS , McManus A , Leung M , Wong P , White MJ . Contextual barriers to lifestyle physical activity interventions in Hong Kong. Med Sci Sports Exerc 2008;40(5):965–71. 10.1249/MSS.0b013e3181659c68 18408599

[33] Fletcher S , Macauley C . The shopping mall as a therapeutic area. Geriatr Nurs 1983;4(2):105–6. 10.1016/S0197-4572(83)80061-5 6550011

[34] Gavin TS . Personal and situational factors affecting exercise involvement [master’s thesis]. Thunder Bay (ON): Lakehead University; 1997.

[35] McCormack GR , Giles-Corti B , Bulsara M . The relationship between destination proximity, destination mix and physical activity behaviors. Prev Med 2008;46(1):33–40. 10.1016/j.ypmed.2007.01.013 17481721

[36] Michael Y , Beard T , Choi D , Farquhar S , Carlson N . Measuring the influence of built neighborhood environments on walking in older adults. J Aging Phys Act 2006;14(3):302–12. 17090807 10.1123/japa.14.3.302

[37] Moore SR . Walking for health: a nurse-managed activity. J Gerontol Nurs 1989;15(7):26–8. 10.3928/0098-9134-19890701-07 2745940

[38] Owens BB . Self-care agency, health promoting lifestyle, and satisfaction with life in postmenopausal women who mall walk. Medsurg Nurs 2007;16(6):383–90. 18390259

[39] Rainey J . Attributions to exercise and adherence to an exercise program [doctoral dissertation]. Columbia (SC): University of South Carolina; 1992.

[40] Schacht SP , Unnithan NP . Mall walking and urban sociability. Sociol Spectr 1991;11(4):351–67. 10.1080/02732173.1991.9981977

[41] Schlapman NJ . Testing the theory of planned behavior in a sample of mall walkers [doctoral dissertation]. Milwaukee (WI): University of Wisconsin; 1994.

[42] Sommers JM , Andres FF , Price JH . Perceptions of exercise of mall walkers utilizing the Health Belief Model. J Health Educ 1995;26(3):158–66. 10.1080/10556699.1995.10603088

[43] Stamford B . Mall walking — burning calories in the great indoors. Phys Sportsmed 1994;22(12):101–2. 10.1080/00913847.1994.11947722 29272965

[44] Standefer CL . The walk of life: an examination of mall-walking and the older woman [doctoral dissertation]. Urbana-Champaign (IL): University of Illinois; 1992.

[45] Stathi A , Gilbert H , Fox KR , Coulson J , Davis M , Thompson JL . Determinants of neighborhood activity of adults age 70 and over: a mixed-methods study. J Aging Phys Act 2012;20(2):148–70. 22472577 10.1123/japa.20.2.148

[46] Stephenson LE , Culos-Reed SN , Doyle-Baker PK , Devonish JA , Dickinson JA . Walking for wellness: results from a mall walking program for the elderly. J Sport Exerc Psych 2007;29:S204.

[47] Travis SS , Duncan HH , McAuley WJ . Mall walking. An effective mental health intervention for older adults. J Psychosoc Nurs Ment Health Serv 1996;34(8):36–8. 8856603 10.3928/0279-3695-19960801-16

[48] Van Puymbroeck M , Hsieh P , Pernell D . Comparison of two physical activity interventions on the physical fitness for informal caregivers. Am J Recreat Ther 2008;7(1):35–41.

[49] Van Puymbroeck M , Hsieh P-C . The influence of mindfulness-based stress reduction and walking on the psychological well-being of female informal caregivers: a pilot study. Am J Recreat Ther 2010;9(1):15–25. 10.5055/ajrt.2010.0002

[50] Waggener GT 3d . Mall walkers: a descriptive study [doctoral dissertation]. Hattiesburg (MS): University of Southern Mississippi; 1993.

[51] Warin M , Moore V , Davies M , Turner K . Consuming bodies: mall walking and the possibilities of consumption. Health Sociol Rev 2008;17(2):187–98. 10.5172/hesr.451.17.2.187

[52] Zenk SN , Wilbur J , Wang E , McDevitt J , Oh A , Block R , Neighborhood environment and adherence to a walking intervention in African American women. Health Educ Behav 2009;36(1):167–81. 10.1177/1090198108321249 18669878 PMC2726823

[53] Belza B , Allen P , Brown DR , Farren L , Janicek S , Jones DL , Mall walking: a program resource guide. Seattle (WA): University of Washington Health Promotion Research Center; 2015. http://www.cdc.gov/physicalactivity/downloads/mallwalking-guide.pdf.

